# Do Not Tell Me More; You Are Honest: A Preconceived Honesty Bias

**DOI:** 10.3389/fpsyg.2021.693942

**Published:** 2021-08-27

**Authors:** David Pascual-Ezama, Adrián Muñoz, Drazen Prelec

**Affiliations:** ^1^Accounting and Financial Administration Department, Universidad Complutense de Madrid, Madrid, Spain; ^2^Sloan School of Management, Massachusetts Institute of Technology, Boston, MA, United States; ^3^RCC Fellow - Harvard Business School, Harvard University, Boston, MA, United States; ^4^Methodology and Social Psychology, Universidad Autónoma de Madrid, Madrid, Spain; ^5^Department of Economics, Massachusetts Institute of Technology, Boston, MA, United States; ^6^Department of Brain and Cognitive Sciences, Massachusetts Institute of Technology, Boston, MA, United States

**Keywords:** dishonesty, cheating, lying, behavioral profiles, detection accuracy

## Abstract

According to the previous literature, only a few papers found better accuracy than a chance to detect dishonesty, even when more information and verbal cues (VCs) improve precision in detecting dishonesty. A new classification of dishonesty profiles has recently been published, allowing us to study if this low success rate happens for all people or if some people have higher predictive ability. This paper aims to examine if (dis)honest people can detect better/worse (un)ethical behavior of others. With this in mind, we designed one experiment using videos from one of the most popular TV shows in the UK where contestants make a (dis)honesty decision upon gaining or sharing a certain amount of money. Our participants from an online MTurk sample (*N* = 1,582) had to determine under different conditions whether the contestants would act in an (dis)honest way. Three significant results emerged from these two experiments. First, accuracy in detecting (dis)honesty is not different than chance, but submaximizers (compared to maximizers) and radical dishonest people (compare to non-radicals) are better at detecting honesty, while there is no difference in detecting dishonesty. Second, more information and VCs improve precision in detecting dishonesty, but honesty is better detected using only non-verbal cues (NVCs). Finally, a preconceived honesty bias improves specificity (honesty detection accuracy) and worsens sensitivity (dishonesty detection accuracy).

## Introduction

Being able to detect when someone is (dis)honest has always been a social goal. A lot of work has been done to identify when people lie and when they tell the truth. In areas like criminology, politics, negotiation, or even playing poker, detecting when someone is lying gives you a competitive advantage over your opponent. It has long been evident in literature that dishonest behavior, both lies (DePaulo et al., 1996) and deception (Weiss and Feldman, 2006), is everyday and frequent occurrences. Therefore, detecting it without the help of technology is essential for everybody in our day-to-day life.

The study of detecting dishonest behavior has come a long way with technology. Truth serums, polygraphs, eye movements, facial analysis, body temperature changes, MRIs, and many other techniques have been used to detect such unethical behavior in the past. More recently, individual physiological responses can offer clues to see dishonest behavior according to contactless non-invasive automatic technologies (also known as automatic deception detection in the literature). Among the different technologies, facial expressions have become one of the most studied features due to their high exposure (e.g., easy to record by a simple camera) and the relevant information of micro-expressions associated with dishonest behavior (e.g., Ekman, 2009). To detect dishonesty, researchers have investigated the potential of automatic physiological approaches, such as a database of facial microexpressions (Pfister et al., 2011) or a method based on dynamic geometric features obtained from facial microexpressions (Owayjan et al., 2012). These earliest approaches demonstrated the capability of automatic systems to detect markers associated with dishonest misconduct. During the last decade, multimodal systems and new machine learning technologies have improved mechanical dishonesty detection performance. Multimodal systems exploit the complementarity of features obtained by a combination of different modalities, such as previously mentioned facial microexpressions, thermal imaging (Rajoub and Zwiggelaar, 2014; Abouelenien et al., 2017), voice (Mendels et al., 2017), and hand gestures (Maricchiolo et al., 2012). In conjunction with available data sets and machine learning algorithms, these multimodal approaches have boosted the performance of automatic systems of wicked recognition accuracy in some scenarios (Krishnamurthy et al., 2018).

However, when technology is not available, no other mechanisms guide us other than our intuition based on our experience to detect the behavior of the person in front of us. Sometimes, when we directly face our opponents, we have environmental or additional information that can help us: something a person has done, something a person has said, or some corporal gesture can give us information and help us have a criterion. It is also possible to ask questions that raise the cognitive load more in liars than in truth-tellers (Vrij et al., 2011). The receptor may likewise become aware of the lie if there are inconsistencies in the message, through verbal (VCs) or non-verbal cues (NVCs), or an investigation after the statement (Ekman, 2009; Vrij et al., 2010). However, many other times, when we only can see the face of the opponent or listen without interaction in the communication, we are able (or we think we are) to detect whether they are honest or dishonest at the time. It is with respect to this situation that we would like to contribute. We want to provide new data on how we are able to detect (dis)honesty when we only see faces of our opponents or when we hear them speak without further environmental interference. With this objective in mind, we put forward the following hypothesis:

*H1: Our ability to detect (dis)honest behavior is directly related to the way we behave (dis)honestly*.

To justify this hypothesis, we will use the existing literature about (dis)honesty detection. There have been two marked trends in the literature, one for and one against, about whether we can detect unethical behavior. There are few studies where we can observe indications that noticing the behavior of others is an elementary, innate ability (e.g., Willis and Todorov, 2006; Fiske et al., 2007; Miller, 2007). Nevertheless, a substantial finding in the deception detection literature indicates that people are not better than casually able to detect a liar (Bond and DePaulo, 2006). So, according to the literature, we should hypothesize that general accuracy will also be no better than chance in our research. However, in addition to analyzing general accuracy, we also want to analyze specificity (honesty detection) and sensitivity (dishonesty detection) since we believe that the ability to detect dishonest people does not necessarily have to be directly related to the ability to detect honest people. To fulfill our purpose, we will sort the literature by answering three fundamental questions: Who can detect dishonesty? How can dishonesty be detected? What information is necessary for detecting dishonesty?

Regarding who can detect (dis)honesty, there is hardly any literature analyzing whether profiles of people who are better able to detect (dis)honesty than others exist. Moreover, there is also no literature dealing with whether those people who are more (dis)honest are better able to detect (dis)honesty. Are dishonest people better at detecting dishonesty than honest people? Are honest people better at detecting honesty than dishonest people? Getting an answer to these questions is the first contribution we wish to make in this research article. With respect to the different profiles of dishonest people, we have the classification proposed by Fischbacher and Föllmi-Heusi (2008, 2013), which offers three types of profiles: honest, liars, and partial liars. In addition, Shalvi et al. (2011) found that when people were allowed to repeat a task more than once but only the first result was valid for reporting purposes, the highest outcome was sometimes reported (even if it was not the first one). Pascual-Ezama et al. (2020) found some additional profiles. In addition to the liars, they found cheater non-liars and radicals. The cheater non-liars did not lie: they reported the result they really obtained, but they obtained the result by repeating the task several times, thus breaking the rules. Even when the rules were strict with respect to doing the task only once (contrary to Shalvi et al., 2011, who permitted the task to be repeated), participants repeated it until they obtained the expected result. On the other hand, radicals reported the result without running the task. They simply reported a result and collected a reward without doing anything. Finally, and in line with Fischbacher and Föllmi-Heusi (2013), Pascual-Ezama et al. (2020) found non-maximizer (partial) profiles for all liars, cheater non-liars, and radicals. Both the strategic behavior of cheater non-liars and the drastic behavior of radicals show two very different patterns of behavior from that of liars. In addition, Pascual-Ezama et al.'s (2020) classification allows us to analyze the data according to four different classifications: first, we consider only whether people are honest or dishonest (simple classification); second, we take into account the different behaviors/strategies of liars, cheater non-liars, and radicals (by nature); third, we consider whether the participants have maximized their dishonesty (by gradient); and finally, we analyze the data according to the eight profiles, two of which are honesty profiles, and six are dishonesty profiles (full classification).

With respect to how dishonesty can be detected, the literature offers evidence for how we can better detect dishonest behavior indirectly, unconsciously (e.g., Reinhard et al., 2013; Brinke et al., 2014), whereas other articles deny this evidence and find the opposite results (see Bond and DePaulo, 2006 for a meta-analysis). Brinke et al. (2014) found some evidence for unconscious lie detection (done without one realizing how), although Franz and von Luxburg (2015), in a critique of the results of the previous study, found evidence for unconscious lie detection but concluded that a significant difference does not imply accurate classification. Moreover, the literature shows that honest behavior (HB) detection is better done with indirect predictions than direct judgments (Vrij et al., 2001; Ulatowska, 2014). It has also been observed that quick, automatic, and subjective decisions make it possible to differentiate between honest and dishonest people much better than premeditated, thoughtful, and objective judgments (DePaulo et al., 2003; Albrechtsen et al., 2009). Taking this information into account and based on the “by nature” classification, we could assert that liars and cheaters are more strategic. They have to think about how they lie or cheat and what strategy they will follow and their decision-making will be more thoughtful and meditated. However, the behavior of radicals will be more automatic, as they do not have to think about their strategy and have clarity regarding what they want to report. Along the same line and based on “by gradient” classification, non-maximizers have a higher self-concept and are less strategic than maximizers, who act in a more meditated manner. Maximizers set their strategy in order to obtain the most money possible, their decision-making being completely objective. However, those who do not maximize due to their self-concept (Mazar et al., 2008) will make their decision-making in an automatic and more subjective way, being an emotional and not very meditated decision. Therefore,

*H1(a): Radicals should be better than cheater non-liars at detecting (dis)honesty*.
*H1(b): Submaximizers should be better than maximizers at detecting (dis)honesty*.

Finally, with respect to what information is necessary to detect dishonesty, there is an extensive literature that analyzes the ability to detect dishonesty in terms of the different cues available, mainly VCs and NVCs. There are widespread beliefs about how people behave when they act dishonesty: stereotypes about gender, ethnicities, or races and about whether dishonest people get nervous and act in a different way. It is also possible to discern information about status, dominance, romantic involvement, and relationship potential (Ambady et al., 2000). There is a general consensus that there has been an overemphasis on NVCs and that VCs are very relevant. One of the most contrasting results in the literature is that the combination of NVCs and VCs is the best way to detect dishonesty. However, the literature is focused on detecting dishonesty but not on detecting honesty. Our second contribution in this paper is to analyze not only dishonesty accuracy detection but also honesty accuracy detection. There is a consensus that VCs facilitate the detection of dishonesty (e.g., Vrij et al., 2010) in two ways: VCs in addition to NVCs (e.g., Ekman and O'Sullivan, 1991; Vrij et al., 2004) and a higher amount of VCs improve accuracy in detecting dishonesty (e.g., Anderson et al., 1999; Feeley and Young, 2000). So, we can affirm that dishonesty is better detected with more information and using VCs. Therefore, we hypothesize that

*H2: Honesty should also be better detected using more information and verbal cues*.

To confirm these hypotheses, we conducted a pilot study with 276 participants, in which we obtained very satisfactory preliminary data, and an experiment with more than 2,000 participants, in which they performed two tasks. The first task consisted of the adaptation of Pascual-Ezama et al. (2020) for the die-under-the-cup task of Fischbacher and Föllmi-Heusi (2013). We decided to use this task, as it is one of the most popular literatures (e.g., Abeler et al., 2019; Charness et al., 2019), With this task, we managed to classify participants according to different profiles of (dis)honesty. The second task consisted of watching a series of TV shows for which participants had to decide whether the contestants were honest or dishonest (other papers used videos: Belot et al., 2012; Serra-García and Gneezy, 2021). In this research paper, we aim to bring more evidence to the literature on detecting dishonest behavior in two ways. On the one hand, we want to examine if different (dis)honest people can detect better/worse (un)ethical behavior of others. We have focused our attention on general accuracy and sensitivity (dishonesty detection accuracy—DDA) and specificity (honesty detection accuracy—HDA) (Baratloo et al., 2015) to determine if different profiles can detect better honesty or dishonesty. On the other hand, we want to analyze if more information and different cues improve not only dishonesty detection but also honesty. Finally, we have detected a bias that makes us overestimate honesty and facilitates the detection of honesty and hinders the detection of dishonesty.

## Experiment

### Materials and Methods

#### Participants

To guarantee enough power in the analyses, we decided to run the experiment with a significant sample of about 2,000 participants. They were finally 2,050 individuals recruited by Amazon Mechanical Turk, who got $1.50 as a show-up fee and the opportunity to earn a $0.50 performance-based bonus in the first part of the experiment. Eighty-seven participants did not complete the task appropriately (did not complete the MTurk process with the MTurk code), so they were eliminated. Another 381 participants were not considered for the analysis, according to the exclusion criterion of Pascual-Ezama et al. (2020)^1^ because they did not follow the rules of the experiment, and therefore we were unable to obtain sufficient information from these participants. The final sample to analyze (dis)honesty detection accuracy consisted of 1,582 participants: 43% were women, and the average age was 37 (SD = 11).

#### Materials and Procedure

Participants ran the experiment on the MTurk platform out of the lab, and they were paid according to their report on the platform. They ran the experiment using the website http://www.behavioralexperiments.com and conducted the experiment in two completely distinct parts. Behavioralexperiments.com is a platform where any researcher can perform experiments. It offers the advantage that it automatically classifies participants according to the classification of Pascual-Ezama et al. (2020) based on their (dis)honesty profiles.

The first part of the experiment consisted of an adaptation of the die-under-the-cup task proposed by Fischbacher and Föllmi-Heusi (2013), using the new paradigm proposed by Pascual-Ezama et al. (2020). Participants were asked to roll the die in http://www.rollandflip.com or a similar website using their cell phone. They would only get no bonus if they got 6, following Fischbacher and Föllmi-Heusi's (2013) rewards system. So, using this task, participants could choose not only to be (dis)honest, but they could adapt it to different levels, from maximum to minimum reward. Every participant received the same message with simple and short instructions: “First, ensure you have a smartphone, a tablet, or another electronic device with internet access. You have to roll a die, and you can earn money depending on your roll result: if you roll a 1, you will receive 0.10$. If you roll a 2, you will receive 0.20$. If you roll a 3, you will receive 0.30$. If you roll a 4, you will receive 0.40$. If you roll a 5, you will receive 0.50$. If you roll a 6, you will receive nothing. Take your cell phone, go to the following website http://www.rollandflip.com/ (or another similar site), select “roll the die” option, and roll the die once.” The critical manipulation here was to link the real outcome and the reported one for a given person. We had access to the rollandflip.com database to match the rolls individually, controlling the exact moment every participant performed the task. Therefore, we were able to determine the precise number of rolls and the real outcome distribution to link with the reported one for each participant. Although not all participants in the study chose to use the rollandflip.com website, most of them did so, allowing us to connect their real and reported outcomes to study honest and dishonest behavior in detail. The website www.rollanflip.com is a website created by researchers to record the real outcome, with the versions “flip the coin” or “roll the die.” We were able to record the real results, IP, timestamp, the reported results, and the time participants took to complete the task. Therefore, we were able to link data from http://www.rollandflip.com with http://www.behavioralexperiments.com to classify real behavior of participants.

In the second part of the experiment, participants had to watch five different videos extracted from the popular TV show in the UK called “golden balls.” In the last part of this program, two contestants have to select between two options. They have two golden balls, one of them has the word “split,” and the other has the word “steal.” If both contestants select split, they share the accumulated money (this varies depending on the evolution of each program). If one contestant selects split, and the other one selects steal, those who select steal obtain all the economic rewards, and the other gets nothing. But, if both contestants choose to steal, both get nothing. This objective of the experiment was to detect whether contestants were honest or dishonest in two different moments. The first moment was before talking (our participants could only see the faces of the contestants, whereas the presenter explained the rules without VCs). In this first moment, participants were asked to give their general opinion on whether they considered the contestants (both) to be honest or dishonest as a general concept. The second moment was after talking; each contestant tried to convince the other to split to open the golden ball with the split/steal option (NVCs + VCs). In this second moment, after hearing the contestants say that they would share the prize (they all do), the participants had to decide whether the contestants were really honest, that means, did they intend to share the prize as they had said (and choose the ball with the word split) or, on the contrary, would they be dishonest, and therefore, despite promising to share the prize, would they choose the steal ball to keep all the money. If the participants decide that a contestant is honest (honesty prediction; HP), and the contestant is honest (HB), the honesty detection (HDA) is considered to be correct. Otherwise, it would be incorrect. Therefore, honesty detection will be the percentage of times a participant detects an honest contestant divided by the total number of contestants who behave honestly (HDA = HP/HB). For example, since the number of dishonest contestants is controlled at 50%, there will be five honest contestants and five dishonest contestants. If a participant detects three of the five honest contestants, they will have an HDA = 3/5 = 60%. Similarly, if the participants decide that a contestant is dishonest (DP) and the contestant behaves dishonestly (DB), the dishonesty prediction (DDA = DP/DB) is considered to be correct. Otherwise, it would be incorrect. Participants also had to answer questions about the two contestants, and they were asked their gender and approximate age before the first question to make sure they did not confuse contestant one and contestant two. We controlled the videos in three ways: the duration of all videos was about 1 min; all participants watched the same videos—five videos with 10 contestants; the contestants were 50% honest and 50% dishonest^2^. We also controlled the race and gender of the contestants to avoid stereotypes. We decided not to financially incentivize this second part of the experiment because it has not been demonstrated that an increase in motivation due to a financial incentive can improve the ability to detect dishonesty. However, we did consider that the pressure to receive an economic incentive could increase anxiety and provoke unnatural decision-making.

### Results

Before presenting the results, we had to be sure to replicate the gray-scale (dis)honesty classification of Pascual-Ezama et al. (2020) with six different dishonesty profiles. We used these profiles to analyze if any profile could detect (dis)honesty better than the others. In Table 1, we can see the profiles found. We used four different models established according to the following classifications: simple classification—taking into account only if people are honest or dishonest; full classification—taking into account the eight profiles, two of which are honesty profiles, and six are dishonesty profiles; by nature, considering the different behaviors/strategies of liars, cheater non-liars, and radicals; and by gradient—taking into account whether the participants maximized their dishonesty. We found all the profiles in this experiment, thus replicating the profiles of Pascual-Ezama et al. (2020).

**Table 1:** Classification of participants according to their reported/actual results.

			**MTurk**	
			**(*n* = 1,582)**	**(*n* = 1,389)**
Roll the die–obtain 5–report 5		Lucky	12.2%	–
Roll the die–obtain x different than 5–report x	Lucky honest	Honest	36.6%	41.7%
Roll the die–obtain 6–report 6	Unlucky honest		8.8%	10%
Roll the die–obtain x different than 5–repeat until x < 5–report x	Submaximizing cheater non-liars	Cheater non-liars	7.8%	8.9%
Roll the die–obtain x different than 5–repeat until 5–report 5	Maximizing cheater non-liars		7.7%	8.8%
Roll the die–obtain x–report > x but < 5	Submaximizing liars	Liars	3.0%	3.4%
Roll the die–obtain x different than 5–report 5	Maximizing liars		5.6%	6.4%
Do not roll the die at all–report < 5	Submaximizing radical dishonest	Radical dishonest	10.3%	11.7%
Do not roll the die at all–report 5	Maximizing radical dishonest		8.0%	9.1%

*Again, gray rows show percentage results, including “Lucky” people. White rows show percentages of the total sample excluding “Lucky” people.

#### Result 1: Submaximizers and Radicals Detect Honesty Better

General accuracy was not different than chance. Participants only guessed correctly about the behavior of the contestants 47% of the time, taking into account its 10 predictions (*p* = 0.5). No differences were found when we repeated the analyses with the simple classification (46 and 47% for honest and dishonest, respectively); when we analyzed by nature, we found 46, 41, 43, and 42%, for honest, liars, cheater non-liars, and radicals, respectively, and by the gradient, the results were 46, 47, and 47%, for honest, submaximizers, and maximizers, respectively. Similar results were found for the full classification. Therefore, and as we might expect according to the literature, the overall predictive ability was absent. We had similar results when we analyzed sensitivity (dishonesty detection) as shown in Table 2.

**Table 2:** (Dis)honesty detection statistics.

**Classification**	***F***	***p***	**η^2^**	**Power**
**Honesty detection accuracy**				
Simple	*F*_(1, 1387)_ = 6.544	0.011	0.005	0.725
By nature	*F*_(1, 1387)_ = 6.887	0.001	0.010	0.923
By gradient	*F*_(1, 1387)_ = 10.389	< 0.001	0.022	0.999
Full	*F*_(1, 1387)_ = 5.458	< 0.001	0.027	0.999
**Dishonesty detection accuracy**				
Simple	*F*_(1, 1387)_ = 0.370	0.847	0.001	0.054
By nature	*F*_(1, 1387)_ = 0.272	0.762	0.001	0.093
By gradient	*F*_(1, 1387)_ = 0.120	0.948	0.001	0.072
Full	*F*_(1, 1387)_ = 0.732	0.645	0.004	0.321

However, when we analyzed specificity (honesty detection), clear differences appeared in the different classifications (see ANOVA in Table 2). In the simple classification (*t*-test), we can see how dishonest people were better at detecting honesty than honest people (64 vs. 60%; *p* = 0.01). By nature, we can observe how radicals were better at detecting honesty than honest people (71 vs. 61%; *p* < 0.001), liars (71 vs. 59%; *p* < 0.001), and cheater non-liars (71 vs. 62%; *p* = 0.013). There were no differences among the rest of the groups. So, this result partially confirms our first hypothesis. Radicals are not better at detecting dishonesty than the rest, but they detect honesty better than any other profile. When we analyzed the data by gradient, we found that submaximizer dishonest people were better at detecting honesty than maximizers (68 vs. 62%; *p* = 0.022) and honest people (68 vs. 61%; *p* < 0.001). This result confirms our second hypothesis. Submaximizers also detected honesty better than any other profile.

#### Result 2: Additional Information Is Not Always Better

Using the single classification, we ran a 2 × 2 ANOVA with level of information (low with NVCs and high with NVCs + VCs) and honesty (honest and dishonest people), and we had two dependent variables: HDA and DDA. In HDA, we found the main effects on level of information and significant interaction but no effects on honesty (see Table 3 for statistics). In DDA, we found the main effects on level of information, but no effects on honesty or interaction. There were significant differences between NVCs and VCs both for dishonest and honest people (both *p* < 0.001), both in HDA and DDA. In HDA, the accuracy of honest people is 61% with NVC and 57% with VC (*p* < 0.001), a similar result to dishonest people (65% NVC vs. 56% VC; *p* < 0.001). Opposite results were found when we analyzed DDA both for honest people (27% NVC vs. 42% VC; *p* < 0.001) and dishonest people (25% NVC vs. 42% VC; *p* < 0.001). When we repeated the analyses using the “by gradient” classification with a 2 × 3 ANOVA with level of information (low with NVCs and high with NVCs + VCs) and honesty (honest, submaximizers, and maximizers), we found similar results. A similar situation arose when we repeated the analyses using the “by nature” classification with a 2 × 4 ANOVA with level of information (low with NVCs and high with NVCs + VCs) and honesty (honest, liars, cheater non-liars, and radicals) (see Tables 3, 4). Therefore, our second hypothesis should be rejected. Honesty is better detected with low levels of information (NVC), whereas dishonesty is better detected with high information levels (NVC + VC).

**Table 3:** Information use statistics.

	***F***	***P***	**η^2^**	**Power**
**HONESTY DETECTION ACCURACY**				
**Simple classification**				
Level of information	*F*_(1, 1387)_ = 63.74	< 0.001	0.044	0.999
Honesty	*F*_(1, 1387)_ = 1.739	0.188	0.001	0.261
Interaction	*F*_(1, 1387)_ = 8.47	0.004	0.006	0.829
**By gradient classification**				
Level of information	*F*_(1, 1387)_ = 70.76	< 0.001	0.049	0.999
Honesty	*F*_(1, 1387)_ = 5.008	0.007	0.007	0.815
Interaction	*F*_(1, 1387)_ = 4.608	0.010	0.007	0.780
**By nature classification**				
Level of information	*F*_(1, 1387)_ = 57.413	< 0.001	0.004	0.999
Honesty	*F*_(1, 1387)_ = 3.905	0.009	0.008	0.829
Interaction	*F*_(1, 1387)_ = 10.706	< 0.001	0.023	0.999
**DISHONESTY DETECTION ACCURACY**				
**Simple classification**				
Level of information	*F*_(1, 1387)_ = 509.892	< 0.001	0.269	0.999
Honesty	*F*_(1, 1387)_ = 1.624	0.203	0.001	0.247
Interaction	*F*_(1, 1387)_ = 2.745	0.098	0.002	0.381
**By gradient classification**				
Level of information	*F*_(1, 1387)_ = 471.874	< 0.001	0.254	0.999
Honesty	*F*_(1, 1387)_ = 1.488	0.226	0.002	0.319
Interaction	*F*_(1, 1387)_ = 1.504	0.223	0.002	0.322
**By nature classification**				
Level of information	[*F*_(1, 1387)_ = 386.796	< 0.001	0.218	0.999
Honesty	*F*_(1, 1387)_ = 1.082	0.355	0.022	0.295
Interaction	*F*_(1, 1387)_ = 3.030	0.028	0.007	0.715

**Table 4:** Information use descriptive.

**HONESTY DETECTION ACCURACY**								
**Simple model**								
Honest (***N*** = 718)		Dishonest (***N*** = 671)						
**NVC**	**VC**	**NVC**	**VC**					
**61%**	**57%**	**65%**	**56%**					
***p*** **< 0.001**	***p*** **< 0.001**							
**By gradient**								
Honest (***N*** = 718)					Submaximizer (***N*** = 334)		Maximizer (***N*** = 337)	
**NVC**	**VC**				**NVC**	**VC**	**NVC**	**VC**
**61%**	**57%**				**68%**	**58%**	**62%**	**54%**
***p*** **< 0.001**					***p*** < 0.001		***p*** < 0.001	
**By nature**								
Honest (***N*** = 718)			Liars (***N*** = 246)		Cheater non-liars (***N*** = 136)		Maximizer (***N*** = 289)	
**NVC**	**VC**		**NVC**	**VC**	**NVC**	**VC**	**NVC**	**VC**
**61%**	**57%**		**59%**	**57%**	**62%**	**54%**	**71%**	**56%**
***p*** **< 0.001**			***p*** < 0.089		***p*** < 0.001		***p*** < 0.001	
**DISHONESTY DETECTION ACCURACY**								
**Simple model**								
Honest (***N*** = 718)		Dishonest (***N*** = 671)						
**NVC**	**VC**	**NVC**	**VC**					
**27%**	**42%**	**25%**	**42%**					
***p*** **< 0.001**	***p*** **< 0.001**							
**By gradient**								
Honest (***N*** = 718)					Submaximizer (***N*** = 334)		Maximizer (***N*** = 337)	
**NVC**	**VC**				**NVC**	**VC**	**NVC**	**VC**
**27%**	**42%**				**24%**	**41%**	**26%**	**43%**
***p*** **< 0.001**					***p*** < 0.001		***p*** < 0.001	
**By nature**								
Honest (***N*** = 718)			Liars (***N*** = 246)		Cheaters non-liars (***N*** = 136)		Maximizer (***N*** = 289)	
**NVC**	**VC**		**NVC**	**VC**	**NVC**	**VC**	**NVC**	**VC**
**27%**	**42%**		**27%**	**41%**	**25%**	**43%**	**22%**	**42%**
***p*** **< 0.001**			***p*** < 0.001		***p*** < 0.001		***p*** < 0.001	

*Bold values indicate the highest between NVC and VC*.

#### Result 3: A “Preconceived Honesty Bias” Is Detected

Specificity (honesty detection) was better than chance both for honest people (58%; *p* < 0.001) and dishonest people (60%; *p* < 0.001), with no difference between submaximizers and maximizers or among liars, cheater non-liars, and radicals. On the other hand, sensitivity (dishonesty detection) was abnormally low again both for honest people (34%; *p* < 0.001) and dishonest people (34%; *p* < 0.001) with no difference between submaximizers and maximizers or among liars, cheater non-liars, and radicals. When we try to detect the behavior of others (dis)honest, we tend to think that honesty prevails, which leads us to have good accuracy in detecting honesty, thinking people are honest. However, we also think that dishonest people are honest, and this leads us to have an extremely poor success rate, much lower than random chance because of a “preconceived honesty bias.”

More evidence to support the “preconceived honesty bias” arose from the difference, both in sensitivity and specificity, with the different levels of information. Having a preconceived bias toward honesty, participants detected honesty very well and dishonesty very poorly with low information. However, as people got more information, they became increasingly hesitant and more likely to think of dishonest behavior, thereby improving sensitivity (26–42%; *p* < 0.001) but significantly worsening specificity (62–56%; *p* < 0.001). Similar results were found for the “by nature” or “by gradient” classifications (see Table 3; *p* < 0.001 for all cases). There was a very pronounced tendency to assume honesty *a priori* when participants only had the visual information of the face of a person (between 22 and 27% in dishonesty detection; *p* < 0.001 for all). This could be a good explanation for why general accuracy is not different than chance at detecting dishonesty, as we can show in our first result and can be found in the literature.

## Discussion and Conclusions

Dishonesty detection is complicated. Even professionals, who work to detect criminal behaviors, perform no better than chance when it comes to detecting dishonesty (e.g., Bond and DePaulo, 2006; Granhag et al., 2015; Serra-García and Gneezy, 2021). The results presented here provide similar results. In line with the literature, our results show how, first, when we try to detect dishonesty, general accuracy is not different than chance, and second, when we increase the amount of information, and VCs, the detection of dishonesty rises considerably although it is still far below chance. We can explain these results from two different points of view. On the one hand, deception could be better detected from multiple cues as has been suggested in many papers that processing a large number of cues could be more efficient (Hartwig and Bond, 2014). On the other hand, in the dishonesty detection literature, people display better performance when using VCs instead of NVCs (e.g., Bond and DePaulo, 2006; Reinhard and Schwarz, 2012). So, results found concerning dishonesty detection are in line with previous results in the literature: we can improve the detection of dishonesty even though it is still far inferior to randomness. However, in analyzing not only general accuracy but also sensitivity (DDA) and specificity (HDA), we discovered a “preconceived honesty bias” to explain these results in the literature.

We have found that results when trying to detect dishonesty just by looking at the face of a person without any other interaction are much lower than those which would correspond to a random outcome. Therefore, the use of basic NVCs not only does not facilitate the detection of dishonesty but also harms it. The literature regarding NVCs and VCs to deception is extensive. There is a consensus that VCs facilitate the detection of dishonesty (e.g., Vrij et al., 2010). Our results show that the natural tendency and predisposition to judge people just by looking at their faces leads us to decide that they are honest. The results repeatedly show that the rate of detection of dishonesty in these circumstances is about 25% when the capacity of random hitting would be double. Therefore, there is a clear “preconceived honesty bias” here that negatively affects the ability of a person to judge our contemporaries at the first glance correctly. However, the vast majority of work has focused on analyzing the ability to detect dishonesty. Still, it has not paid as much attention to the ability (or lack thereof) to detect honesty. In our paper, there are relevant results regarding the cues used for honesty detection. We offer innovative results demonstrating how honesty is well-detected using only NVCs. Again, we can observe the “preconceived honesty bias” in the predisposition to judge people as honest just by looking at their faces. However, more information, in this case, VCs, not only does not improve the ability to detect honesty but significantly worsens it.

The implications of these results are very relevant because if we use only NVCs, we detect honesty better than dishonesty, but with VCs, the contrary occurs. A famous saying is that there is no second chance to make a first impression. In terms of dishonesty detection, our results suggest that to have a correct opinion of our opponent, we should not be guided by that first impression, and we should accumulate more information by combining NVCs and VCs. However, in terms of detecting honesty, the first impression is the correct one. In terms of criminology, a guilty person should remain free in a guaranteed legal system than an innocent person should go to prison. Therefore, we could understand that it would be better to have less information and detect honest people correctly than to stop catching dishonest people. But this logic is not necessarily the right one to apply in the business environment. If we accept that the detection of (dis)honesty is unconscious (done without one realizing how), we have a threshold between detecting more honest or dishonest people. It will depend on the process and on what is of interest at each moment. If we accept that the process is conscious, our results suggest that more research is necessary to understand what information makes our process of detecting honest people worse when we include VCs.

Finally, we found significant results indicating that corrupt people who do not maximize their unethical behavior can detect honesty much better than honest people or dishonest people who maximize their unethical behavior. Submaximizers and radicals are less strategic and act in a more emotional and less meditated manner, so they have a greater critical capacity when establishing their pros and cons of decisions. This situation may mean that they can interpret better the decision-making of the people they observe. They can only do so for honest behavior since dishonest behavior is harder to detect, but they do it much better than the rest. It could also happen that there are hidden variables that we still have not taken into account. For instance, they may be more intelligent either at the level of general intelligence or emotional intelligence, making it easier for them to detect honesty, which is easier to detect than dishonest behavior. This research will be one of the future lines that we will follow. In addition, the perception of contestants of what the counterpart is going to do could be irrelevant in their decision-making. In this case, whatever the reason for their dishonesty, the objective of our participants was to detect whether they would be honest or not, but it is interesting to analyze this situation in another future line of research. But independently of the cause for why submaximizers and radicals can detect better honesty, the fact that they can do it has important implications. In selecting jobs in which honesty is fundamental (casinos, nightlife, security, etc.), submaximizers should conduct interviews. Indeed, they are not honest; still, they are not extremely dishonest either, and their capacity for the correct selection of honest people (above the rest) would imply significant economic benefits. Likewise, they would be much more suitable to carry out negotiation processes since they would regulate the strategies for the profit of company better and better detect their honest behavior of opponents.

## Data Availability Statement

The raw data supporting the conclusions of this article will be made available by the authors, without undue reservation.

## Ethics Statement

The studies involving human participants were reviewed and approved by Universidad Complutense de Madrid. The patients/participants provided their written informed consent to participate in this study.

## Author Contributions

DP-E developed the study concept. Testing and data collection were performed by DP-E and AM. DP-E drafted the manuscript and DP provided critical revisions. All authors contributed to the study design, data analysis and interpretation, and approved the final version of the document for submission.

## Conflict of Interest

The authors declare that the research was conducted in the absence of any commercial or financial relationships that could be construed as a potential conflict of interest.

## Publisher's Note

All claims expressed in this article are solely those of the authors and do not necessarily represent those of their affiliated organizations, or those of the publisher, the editors and the reviewers. Any product that may be evaluated in this article, or claim that may be made by its manufacturer, is not guaranteed or endorsed by the publisher.

## Footnotes

^1^Individuals who gave an immediate response (<5 s after receiving computerized instructions) without using www.rollandflip.com (see the following section) were classified as “radically dishonest” because they claimed the high money outcome without flipping a coin or rolling a die on some other website (5 s is insufficient time to go to an alternative website and/or initiate a coin toss or a die roll). Individuals who gave a report that took more than 5 s and also did not go to our website were eliminated from the final sample.

^2^We repeated the procedure with random selection (70% honest and 30% dishonest contestants) with similar results.
