# Supplementary material for: Do Not Tell Me More; You Are Honest: A Preconceived Honesty Bias
Source: Front Psychol. 2021 Aug 27;12:693942. doi: 10.3389/fpsyg.2021.693942 (PMC8430247; doi:10.3389/fpsyg.2021.693942)
Supplement: Supplementary file 1 [file Data_Sheet_1.pdf]

DIE TASK INSTRUCTIONS

First, ensure you have a smartphone, a tablet, or another electronic device with internet access.

You have to roll a die, and you can earn money depending on your roll result:

1. If you roll a 1, you will receive 0.1$
2. If you roll a 2, you will receive 0.2$
3. If you roll a 3, you will receive 0.3$
4. If you roll a 4, you will receive 0.4$
5. If you roll a 5, you will receive 0.5$
6. If you roll a 6, you will receive nothing.

Take your cellphone, go to the following website http://www.rollandflip.com/ (or another similar site), select "roll the die" option, and roll the die once.

*You will be paid with Mturk's bonus feature.

GOLDEN BALLS INSTRUCTIONS

In this task, you will see several short videos from a game called "Golden Balls".

In Golden Balls, the final round consists of two contestants who must compete for a 'jackpot'. In this jackpot round, each contestant is presented with two balls. One ball has the word 'Split' written inside, and the other has the word 'Steal.' Each contestant has to choose between the two balls knowing that:

If both contestants choose 'Split', they will share the jackpot.
If both contestants choose 'Steal', they each get nothing.

However, if one contestant decides to split and the other to steal, whoever chose to steal will take the entire jackpot, whereas the one who chose to split will get nothing.

You will see a total of 5 videos. Each video is divided into three clips. After the first two clips, you will be asked to indicate whether the contestants seem honest or dishonest (in your opinion). After the third clip, you will be asked to predict which ball (split or steal) you think each contestant will choose.
